# Study of Dispersion, Hydration, and Microstructure of Graphene Nanoplates-Modified Sulfoaluminate Cement Paste

**DOI:** 10.3390/nano12152708

**Published:** 2022-08-06

**Authors:** Kai Cui, Jun Chang, Mohanad Muayad Sabri Sabri, Jiandong Huang

**Affiliations:** 1 School of Civil Engineering, Dalian University of Technology, Dalian 116024, China; 2 Peter the Great St. Petersburg Polytechnic University, 195251 St. Petersburg, Russia; 3 School of Civil Engineering, Guangzhou University, Guangzhou 510006, China

**Keywords:** sulfoalumintae cement, dispersion, microstructure, hydration

## Abstract

Low-carbon ecological cement composites are among the most promising construction materials. With low energy consumption, low carbon dioxide emissions, and high early strength, sulfoaluminate cement (SAC) is a low-carbon ecological building material. In addition, graphene nanoplates (GNPs) exhibit excellent performances. In this study, GNPs were dispersed by a combination of dispersant and ultrasonic treatment, and the dispersion effect of GNPs was characterized. The effect of GNPs on the hydration process and products of SAC was studied, revealing that GNPs accelerate SAC hydration. The hydration heat and ICP results showed that in the SAC hydrolysis stage, C_4_A_3_Š (ye’elimite) hydrolyzed and released Ca^2+^. GNPs absorbed the Ca^2+^, and the Ca^2+^ concentration around C_4_A_3_Š decreased, which would promote the hydrolysis of C_4_A_3_Š and release more Ca^2+^, accelerating the hydration of SAC and the nucleation effect of GNPs, and providing sites for the formation of hydration products. The analysis of XRD (X-Ray Diffraction) and TGA (Thermal Gravity Analysis) showed that GNPs promoted the hydration of SAC and formed more AFt (ettringite) and AH_3_ (gibbsite). The generated hydration products fill the pores of the matrix and are closely connected to the GNPs to form a whole, which improves the cement matrix’s mechanical properties.

## 1. Introduction

More than 4.2 billion tons of cement, the world’s largest artificial building material, is produced annually, emitting a large amount of carbon dioxide; OPC (Ordinary Portland cement) accounts for the majority of this. The amount of carbon dioxide emissions of sulfoaluminate cement is 20–30% lower than that of OPC, and the firing temperature is 200 °C lower than that of OPC. SAC is a low-carbon ecological cement-based material widely used in emergency repair and construction, military engineering, unique engineering, and marine engineering [1,2,3]. With the widespread use of building materials, higher requirements are placed on the performance of materials, and the high brittleness of cement-based materials limits their applications. The development of micro-cracks can lead to the failure of cement-based materials. Therefore, controlling micro-cracks in the matrix is crucial to improving the performance of cement-based materials. Researchers have added steel fibers and nanomaterials to enhance the toughness of the matrix [4,5]. Commonly used nanomaterials are graphene, carbon nanotubes, carbon fibers, nano calcium carbonate, and nano-silica [6,7,8,9,10]; widely used fibers are steel fiber, PVA (Polyvinyl alcohol) fiber, and basalt fiber [11,12,13].

Graphene nanoplates (GNPs) are the basic structural units of graphite materials of all sizes. GNPs are composed of a single layer of carbon atoms wrapped in a two-dimensional honeycomb framework, which can form 0D fullerenes, curled into 1D carbon nanotubes, and stacked into 3D graphite. Compared with other carbon nanomaterials, GNPs have unique advantages. GNPs are two-dimensional nanomaterials with a larger specific surface area than carbon nanotubes [14,15,16,17]. GNPs have excellent mechanical, thermal, and electrical properties; the tensile strength of GNPs is 135 GPa, and their Young modulus is 1.0 TPa, showing better advantages in terms of concrete properties [18,19]. The many advantages of graphene make it a widely used building material [20]. When applying GNPs to concrete materials, the first factor to consider is the dispersion of GNPs. Currently, the commonly used dispersion methods include physical and chemical dispersion. Physical dispersion includes dry mixing, ball milling, and combining the surfactant with ultrasonic dispersion [21]. Chemical dispersion is mainly achieved by changing the atomic structure of graphene and introducing functional groups on GNPs through covalent chemical modification to improve the dispersion of GNPs in water [21]. The oxygen-containing functional groups of graphene significantly enhance the dispersibility of graphene in water, and uniformly dispersed GNPs can improve the mechanical properties of cement-based materials to varying degrees. Many researchers have studied the effect of GO (graphene oxide) on the properties of cement-based materials. Lv et al. found that GO can adjust the hydration products to form a denser microstructure. In addition, it was found that GO can fill the pores of the cement matrix and has a self-healing effect on cracks [22]. Wang et al. studied the mechanism behind GO promoting cement hydration. The oxygen-containing functional groups react with CH (calcium hydroxide) and GO closely embedded in the cement-based material to form a 3D network structure, effectively improving the cement-based composite material’s mechanical properties [23]. Xu et al. showed that GO reacted with hydration products to improve the compressive and flexural strength. The strength improvement is not limited to the advancement of hydration, but GO consumes Ca^2+^ and generates a new hydration product [24]. Zhao et al. investigated the effect of GO on the hydration kinetics, mechanical properties, and microstructure of C-S-H (calcium silicate hydrate). They found that GO could accelerate cement hydration, optimize pore structure, and intercalate into the interlayer space of C-S-H gels through ionic bonding with Ca^2+^ and fill the gel pores [25]. Gao et al. found that GO provided a nucleation point for the hydration reaction and accelerated the hydration of cement [26].

However, GO showed a reduction in the mechanical properties of graphene, including the elastic modulus and tensile strength, and the strengthening and toughening effects are not as sound as those of graphene. Many scholars have also studied the properties of graphene-enhanced cement-based materials. Du et al. found that GNPs could improve the permeability resistance of mortar and reduce the intrusion of water and ions [27]. Vo et al. studied the effect of GNPs with different particle sizes (5 μm, 43 μm, 56 μm, and 73 μm) on cement mortar’s compressive strength and tensile strength, which increased cement hydration due to the effect of van der Waals forces between GNPs [28]. Tang et al. found that a sulfonate graphene nanosheet delayed the dissolution and induction phases of the C3S (Calcium silicate) and hindered the hydration reaction [29]. Yao et al. proposed a method for the in situ growth of graphene on cement particles. The graphene is uniformly dispersed in the cement matrix, promoting the hydration process, forming a denser microstructure, and improving cement-based materials’ mechanical properties [30].

Various studies have shown that GNPs can effectively improve the macro performance of Portland cement-based materials by promoting the hydration process of cement. However, some scholars have also pointed out that GNPs delay the hydration process of cement-based materials and reduce the degree of hydration. Different studies have conflicting conclusions. The hydration mechanism of GNPs when reinforcing cement-based materials has not been entirely determined. There is no sufficient in-depth and systematic scientific explanation and no unified standard for the dispersion and characterization of GNPs. In addition, the current related research is all based on Portland cement-based materials, and there is a lack of research on low-carbon ecological SAC. As we all know, the main mineral compositions of sulfoaluminate cement are C_4_A_3_Š and C_2_S. In the absence of gypsum, C_4_A_3_Š reacts with water and forms AFm (monosulphoaluminate) and AH_3_ (aluminum hydroxide), as shown in Formula (1), this reaction is very rapid and violent. Usually, to control the reaction speed of cement, gypsum is added; in the presence of gypsum, C_4_A_3_Š reacts with water, and gypsum forms ettringite (AFt) and AH_3_; this reaction is shown in Formula (2).

(1) $$C_{4}A_{3}\bar{S}+18H\;\to \;C_{4}A\bar{S}H_{12}+2AH_{3}$$

(2) $$C_{4}A_{3}\bar{S}+2C\bar{S}H_{2}+34H\;\to \;C_{6}A{\bar{S}}_{3}H_{32}+2AH_{3}$$

Based on this, this paper focuses on the effect of graphene after dispersion on the properties of SAC paste. SEM (Scanning Electron Microscope) and TEM (Transmission Electron Microscope) were selected to characterize the dispersion effect of GNPs, and the impact of GNPs on the microstructure of SAC cement-based materials is studied. The effect of the concentration of GNPs on SAC hydration products was analyzed by XRD (X-Ray Diffraction) and TG (Thermal Gravity Analysis). Finally, we explored the underlying mechanism of GNP-enhanced SAC hydration. The results of this study will contribute to future research on using GNPs as additives in cement composites to improve the properties of cement-based materials. Since adding GNPs improves the mechanical properties of cement, it is beneficial to expand the use of SAC. The widespread use of SAC, a low-carbon ecological cement, would be beneficial for reducing carbon dioxide emissions, which is advantageous to the economy and society.

## 2. Materials and Methods

**Figure 1:**
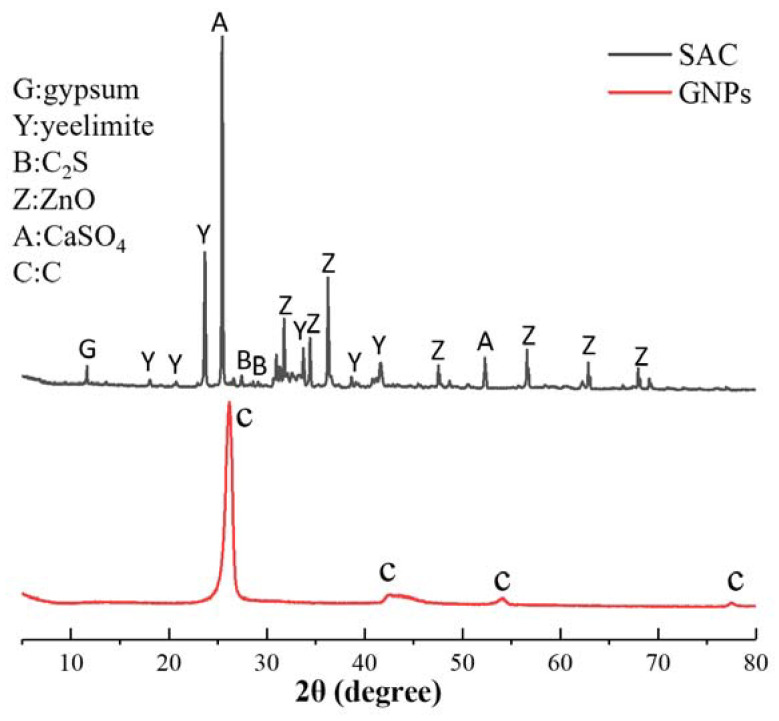
XRD pattern of SAC and GNPs.

GNPs (graphene nanoplatelets), produced by XG science, Inc. (Lansing, MI, USA), the physical parameter is shown in Table 1. SAC (Sulfoaluminate Cement), provided by Tangshan Polar Bear Building Material Co., Ltd. (Tangshan, China). The grade of cement is 42.5. The XRD pattern of SAC and GNPs is shown in Figure 1. The physical properties are shown in Table 2. A polycarboxylate superplasticizer, produced by San sheng Co., Ltd. (Chongqing, China), was used to ensure the workability of cement paste. CO890, provided by Sigma Aldrich, Co. (St. Louis, MO, USA), was used to disperse GNPs. CO890 is a non-ionic surfactant with a hydrophobic group and a hydrophilic group. The hydrophobic group is adsorbed onto the surface of GNPs, and the hydrophilic group penetrates deep into the aqueous solution. It can prevent the intermolecular agglomeration of GNPs through the steric hindrance effect.

The dispersants with different dosages were weighed and poured into a beaker containing 50 mL of deionized water. The mixture was stirred using a glass rod to dissolve the CO890 and subjected to an ultrasonic treatment to disperse the solution. Next, 5 mg of GNPs was poured into the aqueous dispersant solutions under mechanical stirring for 1 min and ultrasonic treatment for 10 min, with an output power of 360 W. Finally; an ultraviolet spectrophotometer was applied to characterize the dispersion effect of GNPs. The water to cement ratio is 0.28 (mass ratio), the dosage values of GNPs are 0.0.06%, 0.09%, and 0.18% of the cement weight, and the sample names are marked as G0, G1, G2, and G3. The dispersion liquid was mixed with SAC, poured into the stirring pot, stirred at low speed for 2 min (rotation speed of 140 ± 5 r/min; revolution speed of 62 ± 5 r/min) and then at high speed for 2 min (rotation speed of 285 ± 10 r/min; revolution speed of 125 ± 10 r/min. Finally, the paste was poured into a 4 × 4 × 16 cm^3^ mold curing for 1d, and the mold was removed and cured for 28 days in a standard curing condition. Finally, the mechanical properties, including compressive and flexural strength, were tested. 

UV-VIS with a wavelength of 260 nm was applied to determine the optimal dosage of dispersant; the microstructure and morphology of GNPs before and after dispersion were observed by FE-SEM (FEI Qunata450450, USA) and TEM (FEI Tecnai G2 F30, USA). The Tam air C80 microcalorimeter (TA instruments company) was used to test the hydration process of SAC with and without GNPs. ICP-OES (Perkin Elmer Optima 2000DV) was used to test the total concentration of Al3+ and Ca2+. Hydration products were measured by XRD (Bruker D8 ADVANCE diffractometer) at a 40 kV and a 40 mA operating current, respectively. EVA software was used to detect hydration products. TG analysis (Mettler Toledo TGA/DSC1, Swiss) was used to detect the hydration sample’s weight loss process at different temperatures. The test temperature ranged from 50 °C to 1000 °C, with a heating rate of 10 °C/min, and the protective gas was nitrogen.

## 3. Results Discussion

### 3.1. Dispersion of GNPs

**Figure 2:**
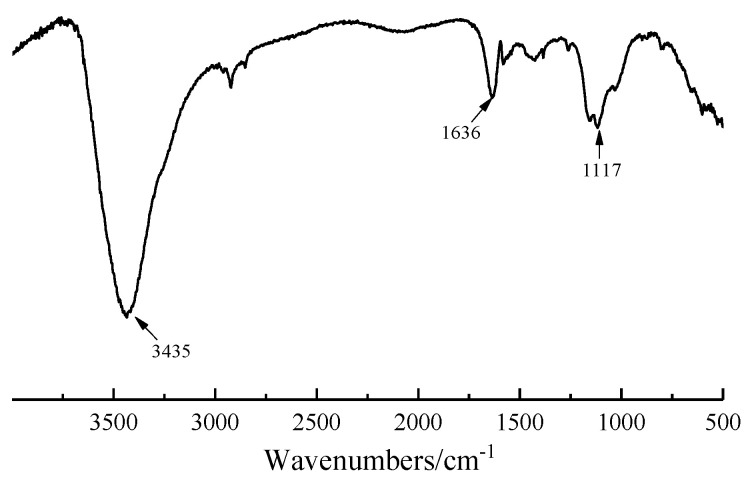
FTIR analysis of GNPs.

**Figure 3:**
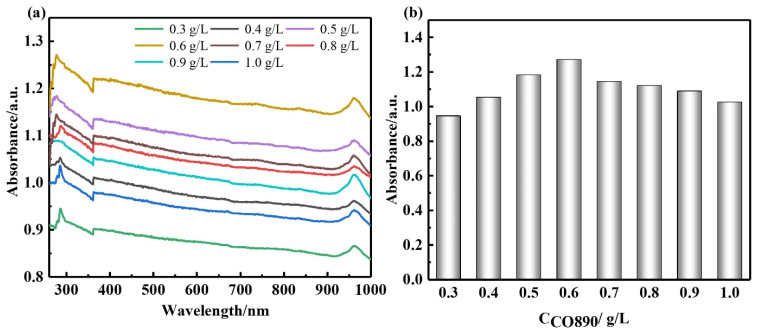
The dispersion of GNPs: (**a**) UV-VIS of GNPs dispersions; (**b**) absorbance of GNP dispersions.

**Figure 4:**
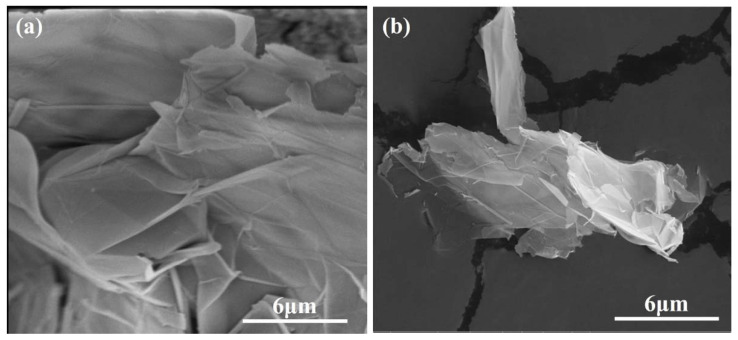
SEM of GNPs: (**a**) before dispersion; (**b**) after dispersion.

**Figure 5:**
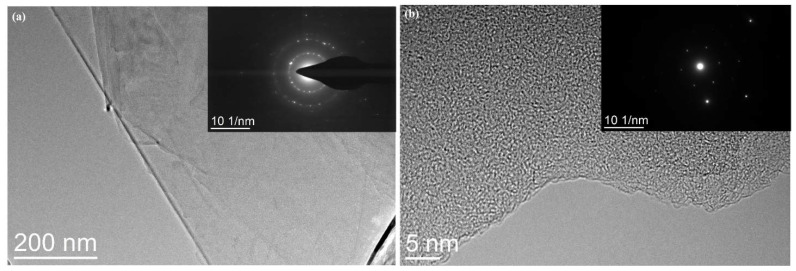
TEM images of GNPs after dispersion.

Figure 1 shows the XRD pattern of GNPs. The diffraction peaks of carbon appeared at 26°, 44°, and 54°. It can be concluded from the diffraction peak intensity that the purity and crystallinity of GNPs were very high. Figure 2 presents the FTIR spectrum of GNPs. GNPs displayed absorption peaks of -OH and -COOH, located at 3435 cm^−1^ and 1636 cm^−1^, respectively. In addition, there was a C-O-C stretching vibration peak at 1117 cm^−1^. According to previous studies [14], CO890 was the dispersant, the concentration of CO890 ranged from 0.3 g/L to 1.0 g/L, and the absorbance of GNPs dispersion liquid with different concentrations was measured. As depicted in Figure 3a, the absorption peak at 260 nm and the absorbance of dispersion liquid showed an increasing trend along with the change of CO890 content. When the concentration of CO890 was 0.6 g/L, as shown in Figure 3b, the absorbance of GNPs reached the maximum value of 1.271. According to the Beer-Lambert Law, we can characterize the dispersion degree of GNPs in the solution. As depicted in Formula (3), GNPs have an excellent dispersion effect. Figure 4a exhibits the morphology of GNPs before dispersion; they have a layered structure and are stacked and twisted, their edges are curled and raised, and their surface is rough. Figure 4b shows an SEM image of GNPs after dispersion; the layered structure of GNPs was thinner, and the curled edges were more open, which was a benefit for bonding hydration products. TEM was used to evaluate the quality of GNPs. Figure 5a,b show the TEM image of GNPs after dispersion. GNPs were multilayered and curled, with wrinkled sheets, indicating that GNPs were very thin. SAED (Selected Area Electron Diffraction) was used to scan the GNPs. From the SAED pattern, as shown in Figure 5a, it was observed that the GNPs were crystalline, indicating that the GNPs were of high quality. From the SAED pattern in Figure 5b, it was observed that the GNPs showed typical hexagonal diffraction spots, indicating single crystal characteristics of GNPs.

(3) $$A=\log(\frac{I_{r}}{I_{s}})=ECL$$

where *E* is a constant, *C* is the solution concentration, and *L* is the optical path length.

According to the literature [31], the actual concentration value of GNPs in the dispersion can be calculated according to the ultraviolet absorbance. It is generally believed that the higher the absorbance value, the higher the actual concentration of GNPs, indicating that the dispersion of GNPs is improved [14].

### 3.2. Macro Performance

**Figure 6:**
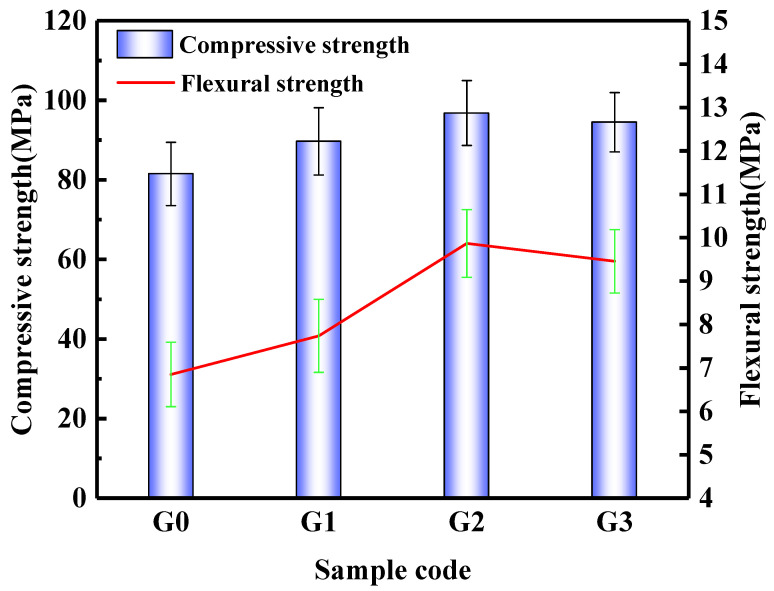
Macro performance of SAC paste.

Figure 6 shows the mechanical properties of the SAC paste. The addition of dispersed GNPs improves the mechanical properties of the SAC paste. The compressive strengths of the G0, G1, G2, and G3 groups after curing for 28d were 81.5 MPa, 89.7 MPa, 96.8 MPa, and 94.5 MPa, respectively. Compared with the G0 group, the compressive strengths of G1, G2, and G3 were increased by 10.1%, 18.8%, and 16.0%, respectively. When the content of GNPs is 0.09%, the compressive strength of SAC pastes at its highest. When the content of GNPs is 0.18%, the growth rate of compressive strength decreases, which may be due to the agglomeration of GNP in the matrix when the content of GNPs is high, therefore reducing the interface bonding between GNPs and the cement paste. The flexural strengths of the G0, G1, G2, and G3 groups after curing for 28d were 6.85 MPa, 7.74 MPa, 9.87 MPa, and 9.46 MPa, respectively. Compared with the G0 group, the flexural strengths of G1, G2, and G3 were increased by 13.0%, 44.1%, and 38.1%, respectively. The changing trend of flexural strength is similar to compressive strength. When the content of GNPs is 0.09%, the flexural strength of SAC pastes at its highest.

### 3.3. Heat of Hydration

**Figure 7:**
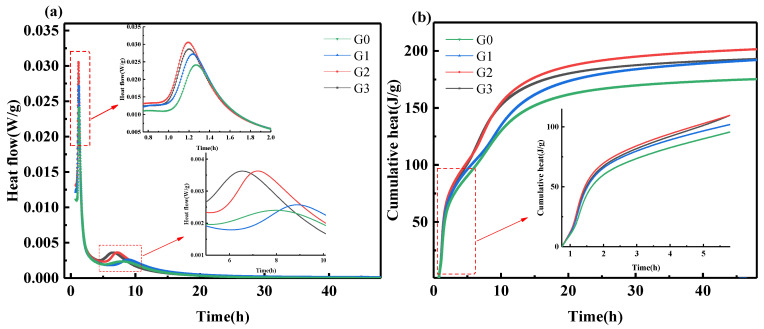
(**a**,**b**) Isothermal calorimetry results.

Figure 7a,b show the effect of GNPs on the hydration heat flow and cumulative heat of SAC. Adding GNPs shortened the induction period of SAC. The induction periods of G0, G1, G2, and G3 ended at 57.3 min, 48.6 min, 54.6 min, and 48 min, respectively, indicating that the acceleration of the hydration of SAC and an increase in the cumulative heat occurred due to the huge specific surface area of GNPs, which provided sites for the nucleation of hydration products. As the hydration reaction proceeded, the first exothermic peak appeared, corresponding to the formation of the AFt. The first exothermic peaks of G0, G1, G2 and G3 appeared at 75.9 min, 74.2 min, 71.5 min, and 72.1 min. The formation of hydration products was closely related to the ion concentration in the solution.

**Figure 8:**
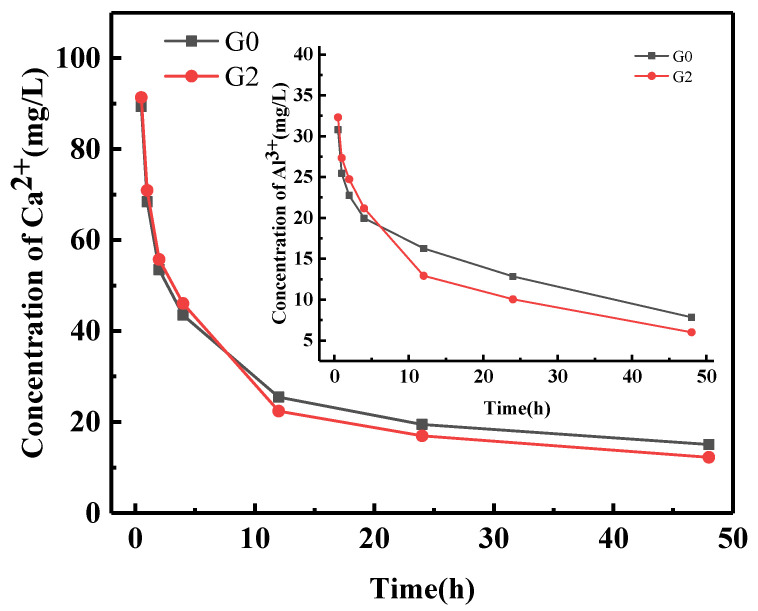
ICP results of sulfoaluminate cement paste.

Figure 8 shows the ion concentration trend of Ca^2+^ and Al^3+^ after hydration samples G0 and G2, measured by ICP. After 0.5 h, 1 h, 2 h, and 4 h of hydration, the Ca^2+^ ion concentrations in the solution of the G0 group were 89.41 mg/L, 68.41 mg/L, 53.47 mg/L, and 43.52 mg/L, respectively. The Ca^2+^ concentrations in the solution of the G2 group were 91.37 mg/L, 70.94 mg/L, 55.75 mg/L, and 46.08 mg/L, respectively. The concentration of Ca2+ in the G2 group was observed to be significantly higher than in the G0 group. The Al^3+^ concentration curve and the Ca^2+^ concentration curve have similar trends.

In the SAC hydrolysis stage, C_4_A_3_Š hydrolyzed and released Ca^2+^. Due to the adsorption effect of GNPs, GNPs absorbed the Ca^2+^, and the Ca^2+^ concentration around C_4_A_3_Š decreased, which promoted the hydrolysis of C_4_A_3_Š and released more Ca^2+^, leading to an increased rate of hydration in SAC and nucleation effect of GNPs, and providing for the formation of hydration products. As the hydration reaction proceeded, the Ca^2+^ and Al^3+^ in the samples decreased. After 12 h, 24 h and 48 h of hydration, the Ca^2+^ ion concentrations in G0 were 25.49 mg/L, 19.48 mg/L, and 15.07 mg/L, and the Ca^2+^ concentrations in G2 were 22.41 mg/L,16.95 mg/L, and 12.26 mg/L, respectively. The concentration of Ca^2+^ ions decreased from 4 h to 12 h due to AFt membrane rupturing due to osmotic pressure and the formation of AFt, corresponding to the second exothermic peak that appeared. AFt and AH_3_ filled the channels as the hydration proceeded, resulting in a higher concentration of Ca^2+^ ions in the G0 group solution than in the G2 group solution. The Ca^2+^ ion concentration in each solution decreased slowly within 12–48 h. 

### 3.4. XRD and TG-DTG Analysis

**Figure 9:**
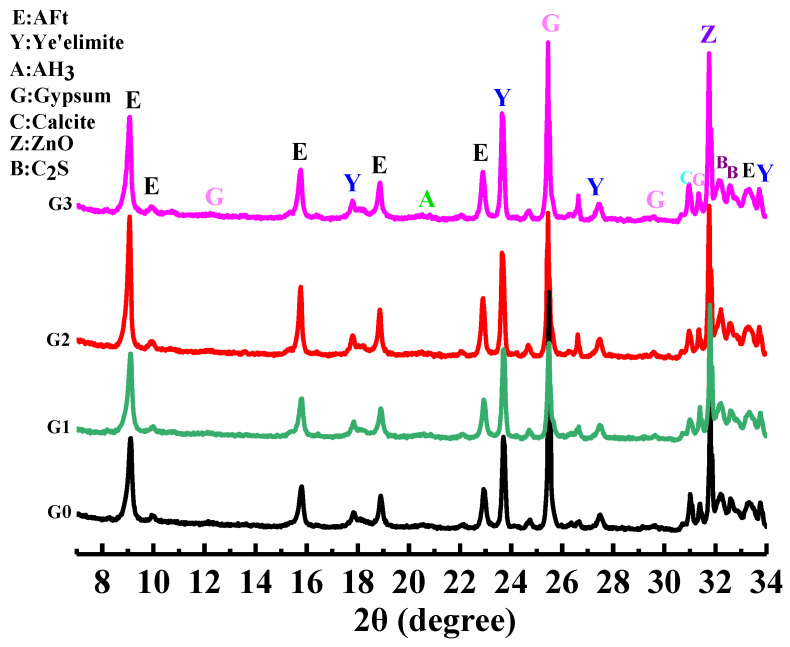
XRD analysis of sulfoaluminate cement paste.

Figure 9 shows the samples’ XRD patterns of AFt, AH_3_, C_4_A_3_Š, C_2_S, and gypsum. It was found that after adding GNPs, the peak positions of different samples were the same, and no new diffraction peaks appeared, but the peak intensities were different. The peak intensity of the AFt in the GNPs group is higher than that of the G0 group, indicating that GNPs promoted the hydration of SAC, which is consistent with other studies that found that the hydration mechanism of GNPs promotes OPC because the nucleation effect of GNPs promoted the hydration reaction. Still, as no chemical reaction occurs, no new hydration products are generated [31].

**Figure 10:**
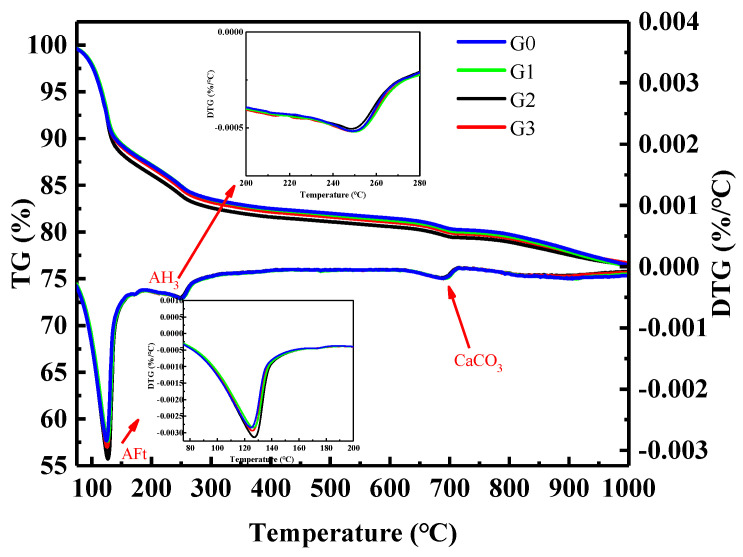
TG−DTG analysis of sulfoaluminate cement paste.

As shown in Figure 10, there were four weight loss peaks. The first weight loss peak appeared at 100–150 °C, corresponding to the dehydration and decomposition of AFt [32,33], and the second peak occurred at 150–200 °C, corresponding to the decomposition of gypsum [32,33]. The third weight loss peak appeared at 210–270 °C [32,33], corresponding to the decomposition of AH_3_. The fourth weight loss peak occurred at 650–800 °C, corresponding to the decomposition of calcium carbonate. In the temperature range of 450–510 °C, there was no weight loss peak, indicating that CH (calcium hydroxide) was not generated, which is consistent with the analysis results of XRD. After adding GNPs, the weight loss of AFt was larger than that of the G0 group, and the TG-DTG and XRD analysis results are consistent. GNPs promoted the hydration of SAC and generated more hydration products.

### 3.5. Reinforcing Mechanism

**Figure 11:**
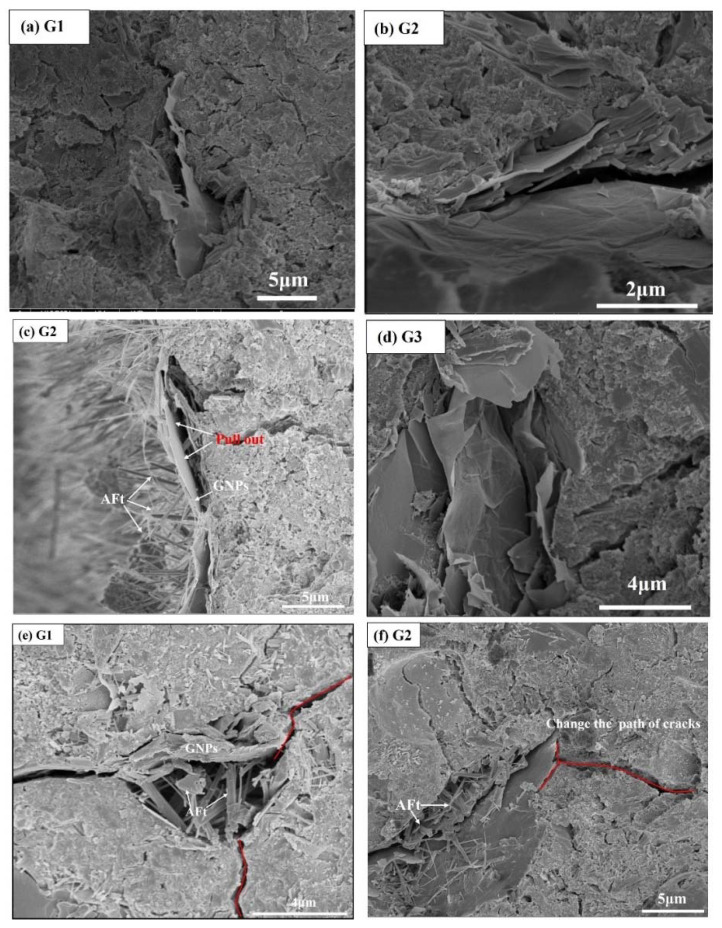
Morphology of GNPs in sulfoaluminate cement paste.

Figure 11 shows the morphology of GNPs in cement-based materials. GNPs are deeply embedded in the cement matrix and are bent and curled. The matrix form a tight bond, which is beneficial to improving the mechanical properties of cement-based materials, as shown in Figure 11a, b. GNPs would be pulled out from the cement matrix when an external force loads the graphene. As shown in Figure 11c, when the GNPs are extracted, GNPs would consume part of the energy and improve the bearing capacity of the matrix. It can be seen that the hydration product AFt is attached to the surface of pulled-out GNPs, indicating that the hydration product and GNPs are tightly connected, further improving the bonding strength between GNPs and the substrate. When the content of GNPs in the cement matrix is too high, GNPs will agglomerate. It can be seen from Figure 11d that the GNPs form parallel planes, and voids form between adjacent GNPs, which are not filled by hydration products. When the load is applied, the parallel planes formed by GNPs are weak, and the GNPs are easily pulled out, thus reducing the mechanical properties. The two-dimensional stacked lamellar structure of GNPs embedded in the cement matrix can bridge and delay the development of microcracks and improve the mechanical properties of the cement matrix, as shown in Figure 11e. When the cracks were filled with GNPs, some were blocked, and some developed to both ends along the GNPs, which significantly increased the cracks’ development paths and improved the matrix’s bearing capacity, as shown in Figure 11f.

From the analysis of hydration heat, ICP, XRD, TG, and SEM, and the changes in macroscopic mechanical properties, we can determine the mechanism of GNPs in promoting SAC hydration, enhancement, and crack inhibition. In the SAC hydrolysis stage, C_4_A_3_Š hydrolyzed and released Ca^2+^. Due to the adsorption effect of GNPs, GNPs absorbed the Ca^2+^ ions, and the Ca^2+^ concentration around C_4_A_3_Š decreased, which would promote the hydrolysis of C_4_A_3_Š and release more Ca^2+^, thus accelerating the hydration of SAC, the nucleation effect of GNPs, and provide sites for the formation of hydration products. GNPs promoted the hydration of SAC and formed more AFt and AH_3_. The generated hydration products fill the pores of the matrix. They are closely connected with the GNPs to form a whole, which improves the cement matrix’s mechanical properties. When the load acted on the matrix, GNPs enhanced matrix performance by filling, bridging, deflecting, and pulling out.

## 4. Conclusions

This paper characterized the dispersion effect of GNPs, studied the effect of GNPs after dispersion on the hydration process and hydration products of SAC, and revealed the mechanism of GNP-enhanced hydration of SAC. Due to the adsorption effect and nucleation effect of GNPs, GNPs were able to absorb Ca^2+^, decreasing the Ca^2+^ concentration around C_4_A_3_Š, which subsequently promoted the hydrolysis of C_4_A_3_Š and release more Ca^2+^, accelerating the hydration of SAC and providing sites for the formation of hydration products. During the hydration process, sufficient ion exchange promoted the hydration of SAC and formed more hydration products, including AFt and AH_3_. These hydration products filled the pores of the matrix. They are closely connected with the GNPs to form a whole, which improves the cement matrix’s mechanical properties. When the load acts on the matrix, the GNPs enhanced the performance of the matrix by filling, bridging, deflecting, and pulling out. This research proposed a new type of low-carbon ecological cement composite, which will help the construction industry to save energy, reduce CO_2_ emissions, and achieve sustainable development. With the discovery and development of the construction industry, GNPs have excellent thermal, mechanical, and electrical properties and can be used in cement to develop intelligent pressure-sensitive materials, electromagnetic wave-absorbing materials, and electromagnetic shielding materials. We believe that, in the future, cement-based smart, multifunctional materials developed with carbon-based materials will become the focus of engineering applications and have broad market prospects.

## Figures and Tables

**Table 1:** Characteristics of GNPs.

Type	Thickness (nm)	Surface Area (m^2^·g^−1^)	Density (g/cm^3^)	Particle Diameters (μm)	Purity (%)
GNPs-M25	6–8	120–150	<3	25	>99.5

**Table 2:** The physical properties of SAC.

Loss on Ignition (%)	Setting Time (min)		Specific Surface Area (m^2^/kg)	Density (g/cm^3^)	Flexural Strength (MPa)		Compressive Strength (MPa)	
	Initial Setting	Final Setting			3 d	28 d	3 d	28 d
1.4	32	56	≥350	3.1	6.3	7.2	42.5	47.2

## Data Availability

The data presented in this study are available on request from the corresponding author.
